# Ultrasound-Guided Sciatic Nerve Block vs. Caudal Analgesia in Cerebral Palsy Undergoing Lower Limb Surgeries

**DOI:** 10.5812/aapm-142479

**Published:** 2023-12-18

**Authors:** Hany Magdy Fahim, Ramy Mahrose, Amr A. Kasem, Mohammed Abdelsalam Menshawi

**Affiliations:** 1 Lecturer of Anesthesia, Intensive Care and Pain Management, Ain Shams University, Cairo, Egypt; 2 Asisstant Professor of Anesthesia, Intensive Care and Pain Management, Ain Shams University, Cairo, Egypt

**Keywords:** Cerebral Palsy, Caudal Block, Subgluteal Sciatic Nerve Block, Postoperative Analgesia

## Abstract

**Background:**

Continuous advancements in ultrasound (US)-guided neuraxial and peripheral nerve blocks (PNB) have allowed the safe and successful use of these blocks as adjuvants to general anesthesia in pediatric patients.

**Objectives:**

This study was designed to compare the analgesic efficacy of 2 US-guided regional techniques, caudal epidural block (CEB) and subgluteal sciatic nerve block (SNB), in children with cerebral palsy (CP).

**Methods:**

The current randomized comparative study was conducted on 30 patients with spastic CP aged 2-12 years who were scheduled for unilateral lower limb multilevel soft tissue corrective surgeries, randomly distributed using a computerized program into 2 equal groups. The CEB group received a US-guided caudal block, and the SNB group received a US subgluteal sciatic nerve block. The time to the first postoperative analgesia requirement (primary outcome), postoperative pain score, total postoperative analgesic consumption, and perioperative complications (secondary outcomes) were assessed in both groups.

**Results:**

The duration of postoperative analgesia was significantly longer in patients of the SNB (14.65 ± 3.08 h) than in the CEB group (5.93 ± 1.68 h). The postoperative pain scores recorded at 6th-12th h and the postoperative 24-h rescue analgesic consumption were significantly lower in the SNB compared to the CEB group.

**Conclusions:**

Ultrasound-guided subgluteal sciatic nerve block is a safe and effective alternative to US-guided caudal analgesia in pediatric patients with spastic CP scheduled for lower limb surgeries, with longer postoperative analgesia and similar perioperative safety profiles.

## 1. Background

Cerebral palsy (CP) is a chronic movement disorder caused by a permanent nonprogressive lesion of the developing brain during the antenatal, perinatal, or postnatal period (1, 2) Spastic CP is the most prevalent type, affecting 70 - 80% of patients with CP. Children with spastic CP often require lower-limb bone and soft tissue corrective surgeries to correct joint deformities and help their ambulation (3-5).

 Children with CP have multiple anesthetic implications due to their various disabilities, such as cognitive impairment, behavioral disturbances, and chronic multisystem dysfunction (6, 7) It is often a major challenge to assess and treat postoperative pain in such populations because of the underlying neurodevelopmental delay, communication difficulties, and vulnerability to the side effects of parenteral opioids (8).

 The caudal block is widely used as an adjunct to general anesthesia in pediatric patients and has been proven to decrease intraoperative anesthetic demands with adequate perioperative pain control (9, 10). The use of peripheral nerve blocks (PNB) in pediatric patients has gained popularity after the incorporation of ultrasound (US) techniques in regional anesthesia practice, with higher rates of successful blocks, using lower doses of local anesthetics, and fewer complications (11-13).

## 2. Objectives

This study was designed to compare the analgesic efficacy of US-guided caudal epidural block (CEB) and subgluteal sciatic nerve block (SNB) in children with spastic CP who underwent lower-limb multilevel soft tissue corrective surgeries.

## 3. Methods

This prospective randomized comparative study was carried out after obtaining ethical committee approval at Ain Shams University Hospitals (reference number FMASU R 24/2023). Written informed consent was obtained from the patients’ parents or legal guardians. This trial was registered at ClinicalTrials.gov (reference number NCT05774132) with an initial registration date of March 17, 2023.

### 3.1. Study Population

Thirty children with spastic CP (American Society of Anesthesiologists (ASA) physical status II) aged 2 - 12 years who underwent elective unilateral lower limb multilevel soft tissue surgeries to correct knee and ankle deformities under general anesthesia in Ain Shams University hospitals from March 20, 2023, to September 27, 2023, were included. Patients were randomly assigned to 2 groups, CEB (caudal, n 15) and SNB (subgluteal sciatic nerve block, n = 15), using a computerized program and in a double-blind manner. For knee flexion deformity, tenotomy of semitendinosus with fractional lengthening of semimembranosus and biceps femoris was performed. Achilles tendon lengthening was performed using Z-plasty for the ankle equinus deformities.

 Patients whose parents refused to provide written informed consent were excluded. Patients with dyskinetic-ataxic or mixed CP, need for corrective surgery for hip contracture/deformity, severe mental disability, active seizure disorders, poor respiratory function, marked renal or hepatic impairment, allergy to amide local anesthetics (LA), infection close to the block injection site, gross sacral deformities, or coagulopathy were also excluded.

### 3.2. Anesthetic Technique

A detailed preoperative anesthesia assessment of all patients, including a history of illness, previous anesthesia, medications, as well as physical examination and airway assessment, was performed. In addition, the laboratory investigations were revised. Instructions for preoperative fasting and continuation of medication were provided to the parents.

 On the day of the operation, patient fasting was ensured, and skin was topicalized with EMLA cream at the suggested site for venous cannulation 1 hour before the surgery. Oral midazolam (0.25 mg/kg) premedication was administered 30 min before transfer to the operating room (OR). The parents were allowed to accompany their children to the OR holding area at which venous access was obtained, and Ringer’s solution infusion was started.

 After transferring the patients to the OR, a 5-lead electrocardiogram (ECG), noninvasive arterial blood pressure monitoring, and pulse oximetry monitoring were initiated. A bispectral index (BIS) monitor and neuromuscular transmission module were used. If intravenous access was secured, premedication with intravenous atropine at a dose of 0.01 mg/kg and ondansetron 0.15 mg/kg was given; then, general anesthesia was induced using fentanyl 1 μg/kg, propofol 1.5 - 2 mg/kg (titrated according to BIS), and rocuronium 0.6 mg/kg. If the child was uncommunicative or combative, inhalational induction was done with sevoflurane 8% in a 50%:50% O_2_: air mixture with a head-up position of 20° to 30° and without positive pressure ventilation while trying to obtain intravenous access, followed by fentanyl, rocuronium, and premedications. A cuffed endotracheal tube was inserted, and the patients were mechanically ventilated to maintain normocapnia. An oropharyngeal temperature probe and a silicone urinary catheter were also inserted.

 Patients were randomized into 2 equal groups by a third party not involved in perioperative patient management or data collection using a computerized program (groups CEB and SNB) according to the planned regional analgesic technique. Great caution was exercised to avoid exceeding the maximum dose of bupivacaine (2 mg/kg) during the LA injection in either group. A linear high-frequency US probe (5 - 13 MHz) was used in all blocks in both study groups and was performed by the same experienced anesthesiologist.

**Figure 1.**
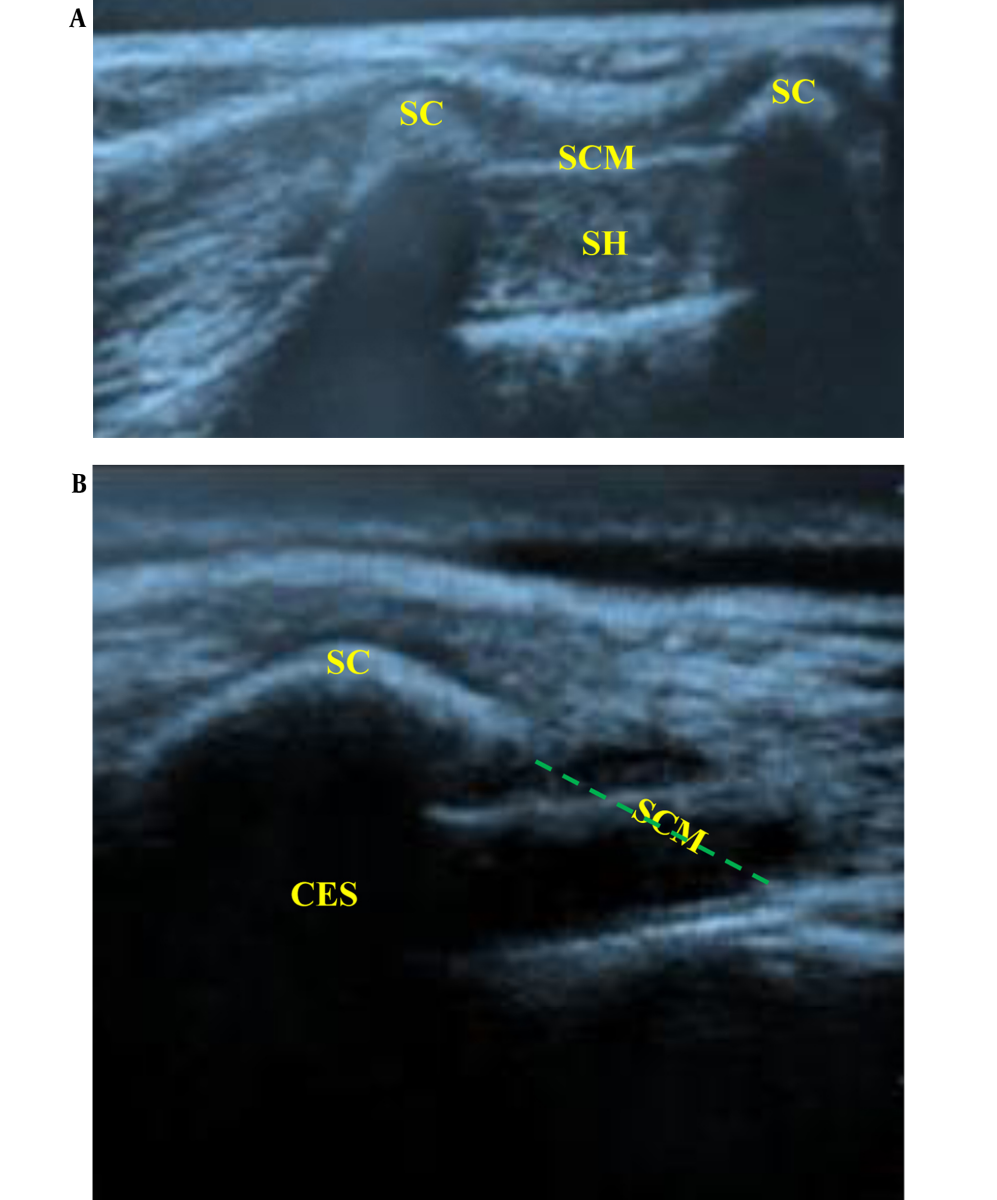
Ultrasound-guided caudal block. (A) Transverse scan of the sacrum at the level of caudal hiatus. Sc (sacral cornuae), SCM (sacrococcygeal membrane), (SH) sacral hiatus (B) longitudinal scan of sacral hiatus while introducing the block needle into the caudal epidural space. CES (caudal epidural space)

In the CEB Group: The patients received US-guided caudal analgesia after anesthesia induction. They were placed in a lateral position with meticulous attention paid to the suspected difficulties caused by pre-existing contractures and limb deformities. After adequate skin disinfection with povidone-iodine, the sacral hiatus was palpated, and the US probe was applied to the sacrococcygeal region in a transverse orientation. The sacral cornua could be identified as 2 hyperechoic reversed U-shaped structures (humps). Between the 2 sacral cornua, 2 hyperechoic lines could be identified; the superficial one was the sacrococcygeal ligament, and the inferior one was the dorsal surface of the sacral bone, while the sacral hiatus was the hypoechoic space between both lines (Figure 1A). An echogenic 22-gauge 5-cm needle was inserted between the sacral cornua into the sacral hiatus utilizing (the out-of-plane approach) to penetrate the sacrococcygeal ligament (pop was felt), which was changed to a longitudinal orientation (90° rotation), and the needle was advanced into the caudal epidural space while visualizing the entire needle length and tip (in-plane approach) (Figure 1B). Careful aspiration was done to confirm the absence of cerebrospinal fluid or blood. Then, 0.1 mL/kg of saline (0.9%) bolus was injected under US guidance to confirm the cranial spread of the injectate in the caudal epidural space while pushing the posterior dura mater anteriorly. Next, LA [bupivacaine 0.25% (1 mL/kg) without exceeding a maximum volume of 20 mL] was injected in increments of 1 mL every 5 seconds under full hemodynamic monitoring.

**Figure 2.**
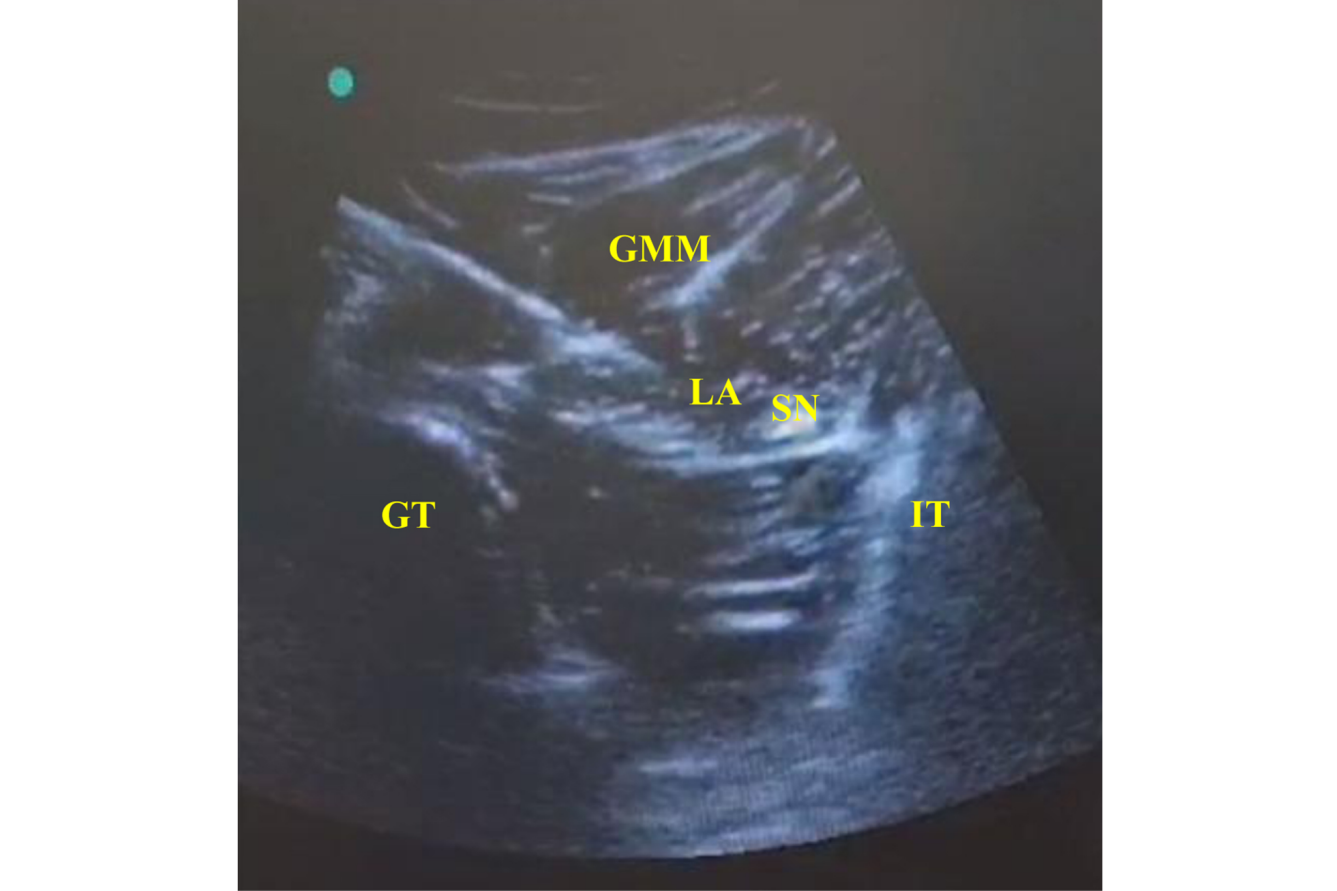
Ultrasound-guided subgluteal sciatic nerve block. The LA was injected to surround the sciatic nerve in the targeted subgluteal plane. SN (sciatic nerve), GT (greater trochanter), (IT) ischial tuberosity, (GMM) gluteus maximus muscle, (LA) local anesthetic

In the SNB Group: The patients received US-guided subgluteal sciatic nerve block after anesthesia induction. Each patient was placed in the lateral position, with the limb at which the nerve block was done in the uppermost position. After adequate skin disinfection with povidone-iodine, the probe was placed at the level of the gluteal crease midway between the 2 bony landmarks, the greater trochanter and ischial tuberosity (hyperechoic lines with acoustic shadowing); the gluteus maximus muscle was identified, and the sciatic nerve was hyperechoic and often elliptical and deep to this muscle (Figure 2). An echogenic 22-gauge 5-cm needle was inserted utilizing the in-plane approach from lateral to medial targeting the sciatic nerve; the LA [bupivacaine 0.25% (0.3 mL/kg) without exceeding a maximum volume of 20 mL] was injected in increments of 1 mL every 5 seconds to surround the whole nerve circumference. Patients’ follow-up after the block (during and after surgery) was performed by an anesthesiologist blinded to the type of regional blockade.

Surgery was started 20 min after caudal or peripheral nerve block. Vital signs were recorded at 5-minute intervals while the blocks were being performed until the end of the surgery. Maintenance of anesthesia was provided with sevoflurane, which was adjusted according to the BIS monitoring targeting its value of 40 - 60, and incremental rocuronium (0.1 mg/kg) according to train-of-four monitoring (TOF). Care was taken during patient positioning, with adequate padding of the bony prominences to avoid pressure sores. All measures were taken to avoid hypothermia by maintaining the OR temperature at 24°C, warming the intravenous (IV) fluids to body temperature, and using warming blankets.

 During surgery, insufficient analgesia was detected by a rise in the heart rate (HR) and mean arterial blood pressure by 20% or more above the baseline recordings. When that occurred, the patient was treated with a rescue dose of intravenous fentanyl (0.5 µg/kg), which could be repeated if required, and such cases were excluded from the study. Intraoperative bradycardia (> 20% decrease in HR below the baseline recordings) was controlled using atropine 0.01 mg/kg IV. When hypotension (MAP dropping below 50 mmHg) was detected, it was controlled by intravenous fluid bolus and incremental ephedrine (0.1 - 0.2 mg/kg) boluses, if needed.

 After the termination of the surgical procedure, the inhalational anesthetic was discontinued, and the reversal of the residual muscle relaxant effect was done using IV neostigmine 0.05 mg/kg with atropine 0.02 mg/kg. Moreover, ETT was removed after the TOF was ≥ 0.9, restoration of gag reflex, and spontaneous eye opening, then the patients were transferred to the post-anesthesia care unit (PACU), where they stayed for the 2 hours observation period.

 In the PACU, the children were allowed to have 1 parent stay with them until discharge. Vital sign monitoring was continued. Chest physiotherapy, suctioning of secretions, and oxygen supplementation were performed as required. After the PACU stay, the patients were admitted to either the surgical floor or the pediatric intermediate care unit at the discretion of the anesthesiologist and surgeon. The patients’ postoperative follow-up and data collection were performed by an anesthesiologist blinded to the type of the regional block received in each study group.

### 3.3. Measured Parameters

-The time to the first postoperative analgesia requirement was measured as the primary outcome.

 -The Revised Face, Leg, Activity, Cry, Consolability (FLACC-R) pain scale score between 0 and 10 (Table 1) (14) was observed and recorded upon arrival to the PACU, every 2 hours for 1st 12 hours postoperatively and every 3 h for the next 12 h postoperatively. Patients with a score of 4 or greater received rescue analgesia by intravenous acetaminophen (perfalgan 15 mg/kg), and if the pain persisted after 15 minutes, another rescue analgesia by ketorolac 0.5 mg/kg IV (slowly) would be given.

**Table 1.** Revised FLACC Pain Scale

Categories	0	1	2
**Individual Behaviors**			
**Face**	No particular expression or smile	Occasional grimace/frown; withdrawn or disinterested; appears sad or worried	Consistent grimace or frown; frequent/constant quivering chin, clenched jaw; distressed-looking face; expression, of fright or panic
**Legs**	Normal position or relaxed; usual tone and motion to limbs	Uneasy, restless, tense; occasional tremors	Kicking, or legs drawn up; marked increase in spasticity, constant tremors or jerking
**Activity**	Lying quietly in a normal position, moves easily; Regular, rhythmic respirations	Squirming, shifting back and forth; tense or guarded movements; mildly agitated (e.g., head back and forth, aggression); shallow, splinting respirations, intermittent sighs	Arched, rigid, or jerking; severe agitation, head banging, shivering (not rigors); breath-holding, gasping, or sharp intake of breaths; severe splinting
**Cry**	No cry/verbalization	Moans or whimpers; occasional complaint; occasional verbal outburst or grunt	Crying steadily, screams or sobs, frequent complaints; repeated outbursts, constant grunting
**Consolability**	Content and relaxed	Reassured by occasional touching, hugging, or being talked to; distractible	Difficult to console or comfort; pushing away the caregiver, resisting care or comfort measures

Abbreviation: FLACC, face, legs, activity, cry, consolability.

- Total postoperative acetaminophen and ketorolac consumption during the first postoperative day

- Incidence of perioperative complications

- Parents’ satisfaction with the postoperative pain management of their children was examined using a 4-point scale (1 = very dissatisfied, 2 = dissatisfied, 3 = satisfied, and 4 = very satisfied).

### 3.4. Sample Size

The primary outcome of this study was the time to the first postoperative analgesia requirement. The sample size was calculated by PASS 15 program, based on the results of a study by Mahrous et al. (15) in which the duration to the first postoperative opioid demand was significantly shorter in the caudal group compared to PNB group, with mean and standard deviation (SD) of 9.6 ± 2.9 vs. 15.1 ± 3.5; considering a 20% dropout rate, the calculated sample size of 15 patients per each study group achieved 97% power to reject the null hypothesis of equal means when the difference between population means is μ1-μ2 = 9.6-15.1 = - 5.5, with SD of 2.9 for 1st group and 3.5 for the 2nd group and a significance level of 0.05, utilizing the 2-samples unequal variance z-test.

### 3.5. Statistical Analysis

 Patients’ data were analyzed using SPSS v. 22 software (IBM Corp., Armonk, NY, USA). Quantitative parametric variables were expressed as mean ± SD, and their intergroup comparison was done using an unpaired t-test. Quantitative nonparametric variables were presented as median (interquartile range), and their intergroup comparison was done using the Mann-Whitney U test. Categorical data were presented as numbers (%), and the chi-square or Fisher’s exact test was used for analysis. P-value indicated statistical significance when below 0.05.

## 4. Results

**Figure 3.**
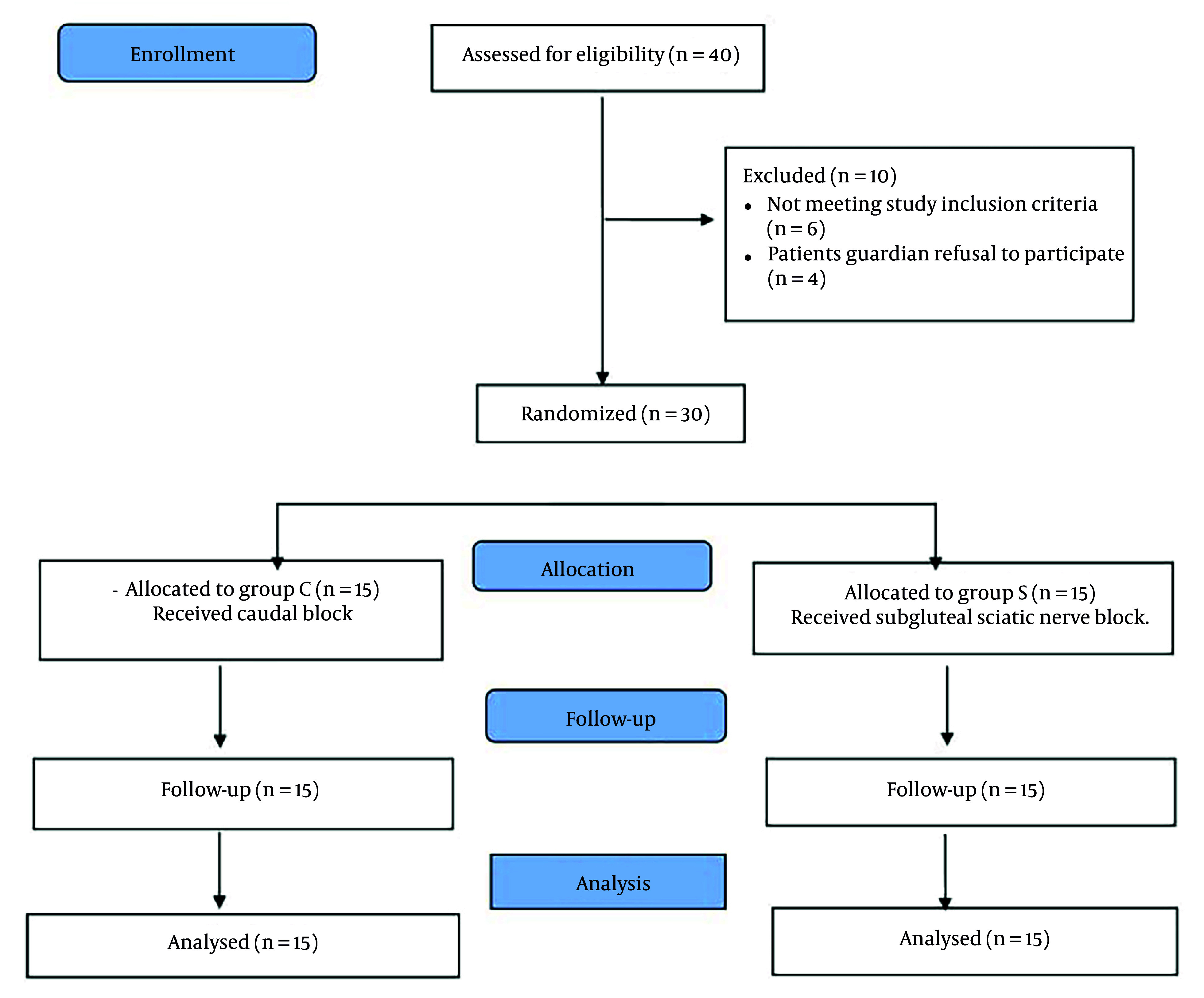
CONSORT flow diagram for the stages of the study

Forty patients with CP who underwent unilateral lower limb multilevel soft tissue surgeries for the correction of knee and ankle deformities were assessed for eligibility in this study. Ten patients were excluded because 4 patients’ guardians refused to participate, and the other 6 patients did not meet the inclusion criteria. Thirty patients were enrolled in this study and blindly randomized into the 2 groups, CEB and SNB, with 15 patients per group. The patients were followed up and analyzed without dropouts (Figure 3). 

The demographic and operative data of the groups indicated that there were no significant intergroup differences (P > 0.05) (Table 2). 

**Table 2.** The Groups’ Demographics and Operative Data ^a^

Variables	CEB (N = 15)	SNB (N = 15)	P-Value
**Age (y)**	6.07 ± 2.68	6.43 ± 3.08	0.739
**Weight (kg)**	18.6 ± 5.31	20.06 ± 6.46	0.517
**Sex (%)**			0.712
Male	9 (60)	8 (53.3)	
Female	6 (40)	7 (46.7)	
**Duration of surgery (min)**	70.46 ± 9.74	73.06 ± 13.29	0.559

^a^ The patients’ data are expressed as means and standard deviations or numbers (%).

 Both caudal and subgluteal sciatic blocks were successfully performed under US guidance. Additional intraoperative opioids were not required in either group. Postoperatively, the revised FLACC scores recorded after PACU arrival at the 2nd, 4th, 15th, 18th, 21st, and 24th postoperative hours revealed no significant intergroup differences (P > 0.05). However, their recordings were significantly lower in SNB in comparison with CEB at the 6th, 8th, 10th, and 12th postoperative hours (P < 0.05) (Table 3). 

**Table 3.** Revised FLACC Score Recordings in Both Groups ^a^

Variables	CEB (N = 15)	SNB (N = 15)	P-Value
**At the PACU**	1 (0-2)	2 (0-2)	0.406
**2h postoperative**	2 (1-2)	1 (0-2)	0.230
**4h postoperative**	2 (1-3)	1 (1-2)	0.204
**6h postoperative**	3 (2-4)	1 (1-2)	< 0.001
**8h postoperative**	3 (2-5)	2 (0-3)	0.004
**10h postoperative**	3 (2-5)	2 (1-3)	0.029
**12h postoperative**	3 (3-4)	2 (1-3)	0.038
**15h postoperative**	3 (2-4)	2 (2-3)	0.271
**18h postoperative**	4(2-5)	3(2-5)	0.417
**21h postoperative**	3(2-5)	3(3-5)	0.849
**24h postoperative**	2 (1-4)	2 (2-3)	0.441

Abbreviation: FLACC, Face, Legs, Activity, Cry, Consolability.

^a^ The patients ‘data are expressed as medians (interquartile range).

The postoperative duration until the first analgesia demand was significantly longer in SNB in comparison with CEB (14.65 ± 3.08 h) versus (5.93 ± 1.68 h) (P < 0.05) (primary outcome). Postoperative paracetamol consumption was significantly less in SNB in comparison with CEB (547 ± 179.39 mg) versus (907 ± 262.44 mg) (P < 0.05). The frequency of rescue ketorolac requirement and total ketorolac consumption 24 h postoperatively was significantly less in SNB in comparison with CEB (P < 0.05) (Table 4). 

**Table 4.** Postoperative Analgesia Requirements in Both Groups ^a^

Variables	CEB (N = 15)	SNB (N = 15)	P-Value
**Postoperative duration to the first analgesia need**	5.91 ± 1.65	14.65 ± 3.08	< 0.001
**Postoperative paracetamol consumption (mg)**	907 ± 262.44	547 ± 179.39	< 0.001
**Frequency of postoperative rescue ketorolac need**	1.93 ± 0.57	1 ± 0.73	< 0.001
**Total postoperative ketorolac consumption**	17.23 ± 5.45	9.76 ± 7.81	0.006

^a^ The patients’ data are expressed as means and standard deviations.

**Figure 4.**
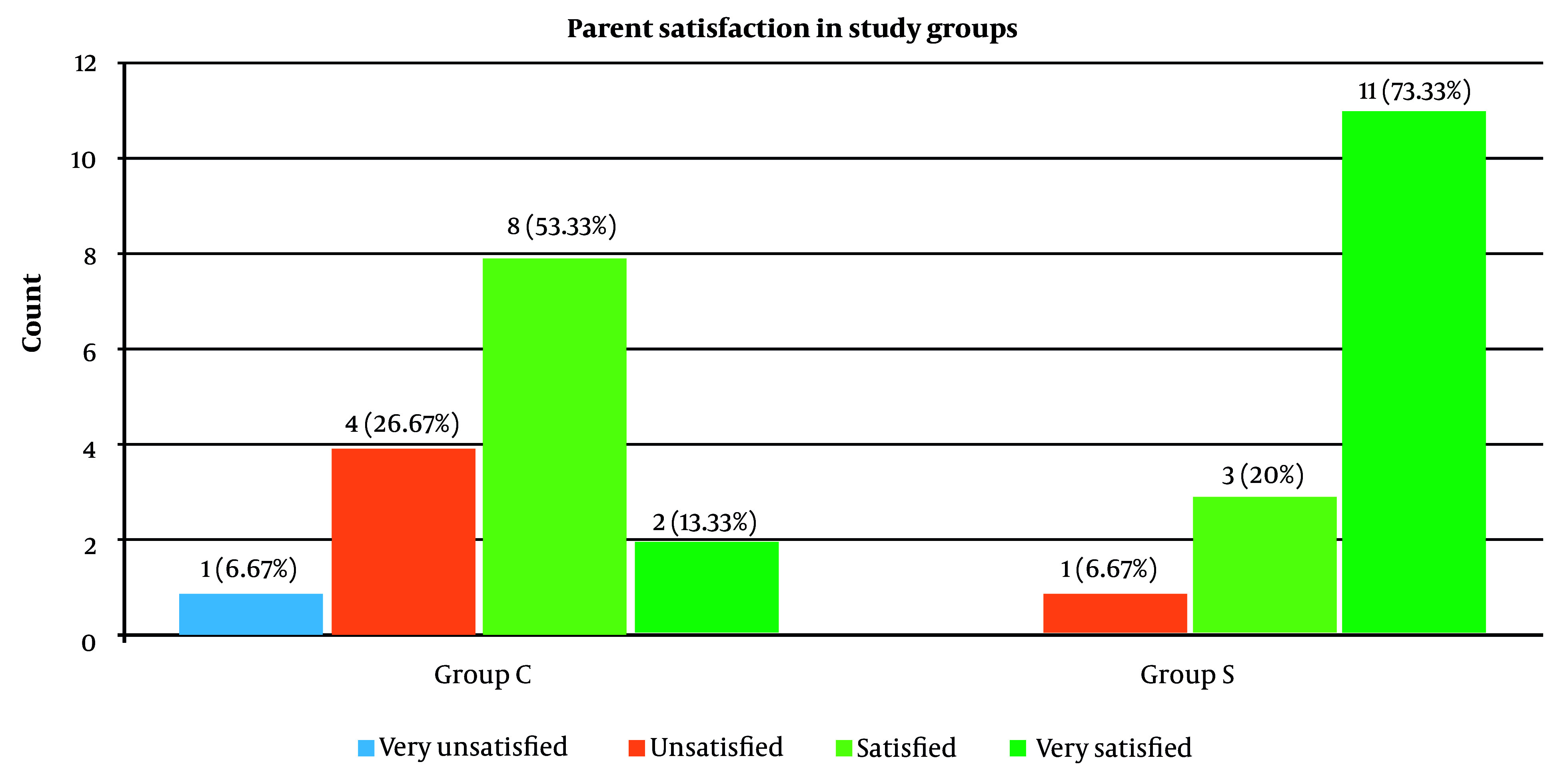
Parents’ satisfaction scores in the groups

Regarding parents’ satisfaction with their children’s postoperative pain management, the majority of the parents in SNB were very satisfied (73.3%), while more than 50% of those in CEB were satisfied (53.3%) (Figure 4). The overall parental satisfaction scores were significantly higher in SNB in comparison with CEB (P <0.001) (Table 5). 

**Table 5.** Parental Satisfaction Scores in Both Groups ^a^

Variable	CEB (N = 15)	SNB (N = 15)	P-Value
** Parental satisfaction scores (1-4)**	3 (2-3)	4(3-4)	0.003

^a^ The patients’ data are expressed as medians (interquartile range).

Regarding the incidence of perioperative complications, there was no significant intergroup difference (P > 0.05). The blocks were performed successfully and uneventfully in both groups without LA anaphylaxis nor systemic toxicity and with no evidence of accidental dural puncture, nerve injury, or hematoma formation. One patient developed intraoperative hypotension in each group, who responded promptly to the bolus of Ringer’s solution. In addition, 1 patient developed laryngospasm after extubation in each group who responded to the jaw thrust maneuver and manual positive pressure ventilation with O_2_ (Table 6). 

**Table 6.** Perioperative Complications in Both Groups ^a,^ ^b^

Perioperative Complications	CEB (N = 15)	SNB (N = 15)	P-Value
** Block-related complications **	0 (0)	0 (0)	-
** Bradycardia**	0 (0)	0 (0)	-
** Hypotension **	1 (6.67)	1 (6.67)	1
** Laryngospasm **	1 (6.67)	1 (6.67)	1
** Vomiting **	0 (0.0)	0 (0)	-

^a^ The patients’ data are expressed as numbers (%).

^b^ Values are presented as No. (%).

## 5. Discussion

Multiple surgical procedures have been described for the correction of lower limb deformities in patients with spastic CP, including soft tissue (tendon) release, lengthening, transfer, and femoral or tibial osteotomies, which can be performed either uni- or bilaterally (3-5). In this study, we chose only 1 type of surgery (soft tissue procedure) to standardize the degree of intensity of painful stimuli that could affect the pain scores recorded postoperatively.

 Both the safety and efficacy of caudal analgesia and PNBs as adjuvants to general anesthesia in the pediatric population were documented in multiple studies, with the advantage of reducing perioperative anesthetic consumption with longer and more effective postoperative analgesia (9, 10, 16-18). Furthermore, the use of US guidance has facilitated the procedure and minimized the incidence of complications (11-13, 19-21).

 To the best of our knowledge, this study is the first one that compared the efficacy and safety of US-guided caudal vs. subgluteal sciatic block in pediatric patients with spastic CP. Our results revealed a significant reduction in postoperative pain scores both at 6th-12th h, longer time to the first postoperative analgesia requirement, and reduction of the overall 24h paracetamol and ketorolac analgesia consumption in the subgluteal sciatic block group compared with the caudal block group.

 The successful application of regional blocks has been reported in patients with CP; epidural blocks have been described in patients with CP to be superior in terms of postoperative pain control compared to systemic (22) or local infiltration analgesia (23). Kim et al. (24) reported the successful use of single-shot caudal analgesia in children with CP scheduled for the repair of the Achilles tendon under general anesthesia. Another study reported the use of a caudal catheter in a CP child with a prior T1 to sacrum fusion scheduled for bilateral lower limb femoral osteotomies under general anesthesia (25).

 Ozkan et al. (26) described the use of US-guided popliteal sciatic nerve blocks in pediatric patients with CP for lower limb orthopedic surgery. They reported a significant reduction in pain scores up to 12 h after surgery, with a significant decrease in total postoperative paracetamol consumption in the block group compared to the control group, with no reported block-related complications. Coppens et al. (27) reported the successful use of a US-guided block for both saphenous and sciatic nerves in a CP patient with an implanted baclofen pump scheduled for bilateral lower limb osteotomies.

 In this study, effective postoperative pain control was achieved in both study groups in the first postoperative 6 hours, whereas afterward (between 6th-12th postoperative hours), postoperative analgesia was significantly better in the subgluteal sciatic nerve block group in comparison with the caudal group. This could be attributed to the limited duration of action of caudal analgesia after a single shot of LA injection, as the epidural space is known for its high vascularity, which accelerates the systemic absorption of LA (28, 29) in comparison with the longer duration of action previously reported with sciatic nerve blocks (14, 26, 30-33).

 In this study, no significant intergroup differences were observed in the incidence of perioperative complications. No block-related complications were reported in either group, especially with direct visualization of the block injection site and real-time injection of the LA under US guidance in all the patients. The safety of both caudal and PNBs in pediatric patients with CP with an upper motor neuron lesion that primarily affects the brain has been reported. (8, 34). Another advantage for both caudal and PNBs in those populations is that they are performed away from the possibly inserted intrathecal baclofen pump that could be exposed to dislodgment or damage with other neuraxial blocks, such as spinal or epidural techniques (27, 34). Also, in patients with CP, previous spinal instrumentation for back deformities, where spinal and epidural techniques are challenging, caudal blocks or PNBs have been described as safe and effective alternatives (25, 34).

### 5.1. Limitations

This study had some limitations. First, it was a single-center design. Second, we did not include a control group in this study because it was considered unethical to perform a placebo injectate or a sham procedure, especially in patients with underlying neurocognitive dysfunction in whom pain should be well-controlled. Third, postoperative pain assessment can be challenging, especially in populations with underlying cognitive impairment. To minimize bias, we used the r-FLACC scale, which is proven to be a valid and reliable measure to assess pain in the pediatric population with variable degrees of cognitive impairment (35, 36). Fourth, we used single-shot blocks for both caudal and PNB without using adjuvants or inserting a catheter, which could be useful for extending postoperative analgesia that could be further evaluated.

### 5.2. Conclusions

From the results obtained in the current study, it can be concluded that US-guided subgluteal sciatic nerve block is a safe and effective alternative for US-guided caudal analgesia in pediatric patients with spastic CP scheduled for lower limb surgeries, with longer postoperative analgesia and similar perioperative safety profiles.

## Clinical Trial Registration Code:

This trial was registered at ClinicalTrials.gov (reference number NCT05774132) with an initial registration date of March 17, 2023.

## References

[1] Stavsky M, Mor O, Mastrolia SA, Greenbaum S, Than NG, Erez O (2017). Cerebral palsy-trends in epidemiology and recent development in prenatal mechanisms of disease, treatment, and prevention.. Front Pediatr..

[2] Shaikh SI, Hegade G (2017). Role of anesthesiologist in the management of a child with cerebral palsy.. Anesth Essays Res..

[3] Gupta A, Srivastava A, Taly AB, Murali T (2008). Single-stage multilevel soft-tissue surgery in the lower limbs with spastic cerebral palsy: Experience from a rehabilitation unit.. Indian J Orthop..

[4] Hosangadi A, Varma A, Kalluraya S (2018). Lower extremity soft tissue surgery in spastic cerebral palsy: Experience from a government rehabilitation unit.. International J Res Orthopaedics..

[5] Mu X, Deng B, Zeng J, Zhang H, Zhao Y, Sun Q (2020). Orthopedic treatment of the lower limbs in spastic paralysis.. Brain Science Advances..

[6] Shrader MW, Jones J, Falk MN, White GR, Burk DR, Segal LS (2015). Hip reconstruction is more painful than spine fusion in children with cerebral palsy.. J Child Orthop..

[7] Allen J, Zareen Z, Doyle S, Whitla L, Afzal Z, Stack M (2021). Multi-organ dysfunction in cerebral palsy.. Front Pediatr..

[8] Sadacharam K, Brislin RP, Lang R (2018). Regional anesthesia in patients with cerebral palsy.. Cerebral Palsy..

[9] Benka AU, Pandurov M, Galambos IF, Rakic G, Vrsajkov V, Draskovic B (2020). [Effects of caudal block in pediatric surgical patients: A randomized clinical trial].. Braz J Anesthesiol..

[10] Sanghvi C, Dua A (2023). Caudal Anesthesia.. StatPearls..

[11] Guay J, Suresh S, Kopp S (2017). The use of ultrasound guidance for perioperative neuraxial and peripheral nerve blocks in children: A cochrane review.. Anesth Analg..

[12] Merella F, Mossetti V (2020). Ultrasound-guided upper and lower extremity nerve blocks in children.. BJA Educ..

[13] Catalani B, Jones JJ (2022). Peripheral nerve block complications in children.. Orthop Clin North Am..

[14] Malviya S, Voepel-Lewis T, Burke C, Merkel S, Tait AR (2006). The revised FLACC observational pain tool: Improved reliability and validity for pain assessment in children with cognitive impairment.. Paediatr Anaesth..

[15] Mahrous RSS, Ahmed AAA, Ahmed AMM (2022). Comparison between ultrasound-guided caudal analgesia versus peripheral nerve blocks for lower limb surgeries in pediatrics: A randomized controlled prospective study.. Local Reg Anesth..

[16] Suresh S, Long J, Birmingham PK, De Oliveira GJ (2015). Are caudal blocks for pain control safe in children? an analysis of 18,650 caudal blocks from the Pediatric Regional Anesthesia Network (PRAN) database.. Anesth Analg..

[17] Kendall MC, Alves LJC, Suh EI, McCormick ZL, De Oliveira GS (2018). Regional anesthesia to ameliorate postoperative analgesia outcomes in pediatric surgical patients: An updated systematic review of randomized controlled trials.. Local Reg Anesth..

[18] Walker BJ, Long JB, Sathyamoorthy M, Birstler J, Wolf C, Bosenberg AT (2018). Complications in pediatric regional anesthesia: An analysis of more than 100,000 blocks from the pediatric regional anesthesia network.. Anesthesiology..

[19] Greaney D, Everett T (2019). Paediatric regional anaesthesia: Updates in central neuraxial techniques and thoracic and abdominal blocks.. BJA Educ..

[20] Ponde V (2019). Recent trends in paediatric regional anaesthesia.. Indian J Anaesth..

[21] Khan ZH, Karvandian K, Zanjani AP (2017). Ultrasound Guided Peripheral Nerve Blocks in Pediatric Patients on The Basis of Anatomical Areas; Upper and Lower Extremities: A Narrative Review.. Arch Anesth & Crit Care..

[22] Moore RP, Wester T, Sunder R, Schrock C, Park TS (2013). Peri-operative pain management in children with cerebral palsy: Comparative efficacy of epidural vs systemic analgesia protocols.. Paediatr Anaesth..

[23] Kjeldgaard Pedersen L, Nikolajsen L, Rahbek O, Uldall Duch B, Moller-Madsen B (2016). Epidural analgesia is superior to local infiltration analgesia in children with cerebral palsy undergoing unilateral hip reconstruction.. Acta Orthop..

[24] Kim SH, Chun DH, Chang CH, Kim TW, Kim YM, Shin YS (2011). Effect of caudal block on sevoflurane requirement for lower limb surgery in children with cerebral palsy.. Paediatr Anaesth..

[25] Dixit D, Theroux MC, Dabney KW, Miller F (2018). Use of caudal epidural catheter in a child with cerebral palsy with prior posterior spine (T1-sacrum) fusion.. Indian J Anaesth..

[26] Ozkan D, Gonen E, Akkaya T, Bakir M (2017). Popliteal block for lower limb surgery in children with cerebral palsy: Effect on sevoflurane consumption and postoperative pain (a randomized, double-blinded, controlled trial).. J Anesth..

[27] Coppens S, Hoogma D, Rex S, Van De Velde M (2019). Bilateral popliteal and saphenous nerve block for osteotomy and tendon release in a cerebral palsy patient with intrathecal baclofen pump: A case report.. Acta Anæsthesiologica Belgica..

[28] Engelman E, Marsala C (2012). Bayesian enhanced meta-analysis of post-operative analgesic efficacy of additives for caudal analgesia in children.. Acta anaesthesiologica Scandinavica..

[29] Narasimhan P, Kashyap L, Mohan VK, Arora MK, Shende D, Srinivas M (2019). Comparison of caudal epidural block with paravertebral block for renal surgeries in pediatric patients: A prospective randomised, blinded clinical trial.. J Clin Anesth..

[30] Oberndorfer U, Marhofer P, Bosenberg A, Willschke H, Felfernig M, Weintraud M (2007). Ultrasonographic guidance for sciatic and femoral nerve blocks in children.. Br J Anaesth..

[31] van Geffen GJ, Pirotte T, Gielen MJ, Scheffer G, Bruhn J (2010). Ultrasound-guided proximal and distal sciatic nerve blocks in children.. J Clin Anesth..

[32] Dillow JM, Rosett RL, Petersen TR, Vagh FS, Hruschka JA, Lam NC (2013). Ultrasound-guided parasacral approach to the sciatic nerve block in children.. Paediatr Anaesth..

[33] Halpern L, Kogan CJ, Arnzen G (2022). Peripheral Nerve Blockade for Medial Patellofemoral Ligament Reconstruction in Pediatric Patients: The Addition of a Proximal Single-Injection Sciatic Nerve Block Provides Improved Analgesia.. Local Reg Anesth..

[34] Cung S, Ritz ML, Masaracchia MM (2021). Regional anesthesia in pediatric patients with preexisting neurological disease.. Paediatr Anaesth..

[35] Pedersen LK, Rahbek O, Nikolajsen L, Moller-Madsen B (2015). The revised FLACC score: Reliability and validation for pain assessment in children with cerebral palsy.. Scand J Pain..

[36] Sierra‐Núñez D, Zuriguel‐Pérez E, Bosch‐Alcaraz A (2022). Postsurgical pain assessment in children and adolescents with cerebral palsy: A scoping review.. Dev Med Child Neurol..

